# Automatic Classification of Rotor Faults in Soft-Started Induction Motors, Based on Persistence Spectrum and Convolutional Neural Network Applied to Stray-Flux Signals

**DOI:** 10.3390/s23010316

**Published:** 2022-12-28

**Authors:** Vicente Biot-Monterde, Angela Navarro-Navarro, Israel Zamudio-Ramirez, Jose A. Antonino-Daviu, Roque A. Osornio-Rios

**Affiliations:** 1 Instituto Tecnológico de la Energía (ITE), Universitat Politècnica de València (UPV), Camino de Vera S/N, 46022 Valencia, Spain; 2 HSPdigital CA-Mecatronica Engineering Faculty, Autonomous University of Queretaro, San Juan del Rio 76806, Mexico

**Keywords:** induction motor, CNN, stray-flux, automatic fault diagnosis, soft starters, broken rotor bars

## Abstract

Due to their robustness, versatility and performance, induction motors (IMs) have been widely used in many industrial applications. Despite their characteristics, these machines are not immune to failures. In this sense, breakage of the rotor bars (BRB) is a common fault, which is mainly related to the high currents flowing along those bars during start-up. In order to reduce the stresses that could lead to the appearance of these faults, the use of soft starters is becoming usual. However, these devices introduce additional components in the current and flux signals, affecting the evolution of the fault-related patterns and so making the fault diagnosis process more difficult. This paper proposes a new method to automatically classify the rotor health state in IMs driven by soft starters. The proposed method relies on obtaining the Persistence Spectrum (PS) of the start-up stray-flux signals. To obtain a proper dataset, Data Augmentation Techniques (DAT) are applied, adding Gaussian noise to the original signals. Then, these PS images are used to train a Convolutional Neural Network (CNN), in order to automatically classify the rotor health state, depending on the severity of the fault, namely: healthy motor, one broken bar and two broken bars. This method has been validated by means of a test bench consisting of a 1.1 kW IM driven by four different soft starters coupled to a DC motor. The results confirm the reliability of the proposed method, obtaining a classification rate of 100.00% when analyzing each model separately and 99.89% when all the models are analyzed at a time.

## 1. Introduction

Induction Motors (IMs) are widely used in a large part of industrial applications in industrialized countries [1]. Their robustness, reliability, easy maintenance and low cost, among other characteristics, have contributed to this fact. Squirrel Cage Induction Motors (SCIM), more specifically, are a significant part of the IMs used in those applications [2], consuming almost 89% of the power that industrial facilities demand [3]. Despite those characteristics, SCIM are not immune to failures. Due to the high currents during the start-up and other transients, they deal with thermo-mechanical stresses in the rotor bars that can lead to a fault. This is particularly true in applications where continuous cycles of start–stop are required [4]. To avoid stresses during the start-up, several starting systems are used in the industry. Among others, the use of auto-transformers, stator resistors, soft starters or the star-delta starting are the most usual starting systems [5]. In this context, soft starters have become one of the most preferred starting systems due to their advantages. By means of a power electronics circuit, based typically on thyristors connected in anti-parallel and installed on one-, two- or the three-supply phases, these devices allow one to limit the start-up currents. Changing the conduction time of the thyristors, the Root Mean Square (RMS) value of the supply voltage can be modified, hence modifying the profile of the starting current. These devices allow, also, to control the duration of the voltage ramp and the initial voltage of the supply during the starting of the motor. Some models, even allow to set a maximum value for the starting current. Nevertheless, although the use of these devices reduces stresses in the motors during the starting, it has been proven in some works that this does not eliminate the risk of failure. Indeed, the electronics in soft starters amplify certain harmonics and introduce new ones that, in some cases, can lead to additional stresses in the rotor [6,7,8,9]. Moreover, secondary torque harmonics can appear, resulting in resonance problems [10].

On the other hand, although motors with rotor failures like broken bars can keep running, their efficiency decreases, resulting in a higher energy consumption and hence, higher energy costs [11]. Furthermore, their life expectancy will decrease, and unexpected catastrophic failures could occur, due to the fact that this kind of failure does not reveal external symptoms of existence in its initial stages [2,12]. As a consequence of this, the processes depending on the machine that fails can suffer shutdowns, generating, depending on the application, high economical and time losses [13]. It is because of this that in recent years, high efforts in developing new condition monitoring methodologies for electrical motors have been made. The aim of these methodologies is to detect the different motor faults in their initial stages, allowing, consequently, to avoid unexpected costs and catastrophic failures. In general terms, these techniques rely on extracting information from a motor’s different physical quantities (vibrations, stray-flux, currents, temperatures, partial discharges, etc.), once captured by means of proper sensors. That information is obtained by applying advanced signal processing tools (STFT, FFT, DWT, etc.). Although after all these years of research in this area it is accepted that no method based on analyzing a single physical magnitude can determine the health state of the whole motor [14], the analysis of a specific magnitude may be much suitable to detect some specific failures than others [15]. In this regard, the analysis of the stray-flux during the start-up transient is an effective method to detect the presence of broken bars in the rotor of induction machines. The higher harmonic content in the resulting time–frequency maps compared to that obtained in the analysis of currents enables a more reliable diagnosis of this specific failure [16]. Another conclusion that arises from [14] is that the development of intelligent diagnosis systems is a current trend in this area of research. In this regard, many previous works have applied several techniques to identify and classify rotor faults in induction motors, but near all of them are referred to Direct Online (DOL) started motors [17]. For instance, in [18] the authors applied a (Convolutional Neural Network) CNN to diagnose rotor bar breakages in DOL-started induction motors, achieving an accuracy rate of 66.7%. In this case, images of the STFT time–frequency (t–f) maps of the stray-flux were used. On the other hand, in [19], the authors used t–f maps obtained from current signals of a DOL induction motor to identify the typical patterns of bar breakages, reaching an accuracy rate of 97.5%. In [20], the authors proposed the use of a Feed-forward Neural Network (FFNN) to analyze the stray-flux signals obtained from a DOL induction motor, obtaining an accuracy rate of 97%. In addition, in [21], the authors applied also an FFNN, but this time, to a combination of stray-flux and current signals, achieving an accuracy rate of 95%. Other works, like [22,23,24], used CNN for the detection of bar breakages in IMs, obtaining accuracy rates of 100%, 100% and 97.87%, respectively, but all the cases applied to DOL-started motors.

On the other hand, as it was said before, the use of soft starters introduces new harmonics in the current and stray-flux signals. In some previous works, it has been proven that, because of this fact, the identification of the fault patterns with some time–frequency tools becomes more difficult using both currents [7,8] and stray-flux [25,26]. Thus, it is important to obtain new methods that could lead to automatically identifying the presence of these faults, before a catastrophic failure occurs, when soft starters are used. In this regard, in [27], the authors proposed the use of a CNN, applied to the stray-flux signals, as a method to detect the presence and the severity of bar breakages in induction motors driven by soft starters. The accuracy rate achieved in this work was 94.40%. By their side, in [28], the authors used Linear Discriminant Analysis (LDA) and an FFNN, applied to a combination of current and stray-flux signals, to detect the presence and the severity of bar breakages in an IM driven by soft starters. In this case, the accuracy rate achieved was 94.40%.

Attending to all the above-mentioned considerations, the automatic methods for rotor fault detection in soft-started induction motors are still improvable. This work presents a new methodology for the automatic detection and severity categorization of rotor faults in induction motors driven by soft starters. The novelty of the proposed methodology is the use of the Persistence Spectrum (PS) applied to the start-up transient stray-flux signals. Then, a convolutional neural network (CNN) is used to automatically categorize the severity of the rotor faults. In order to improve the dataset, data augmentation techniques are used. In this regard, Data Augmentation Techniques (DAT) have been proven to be a reliable way to enhance the data base used in CNN. In [18,27] it was stated that the use of Data Augmentation Techniques is a reliable method to deal with the scarcity of samples, providing a good dataset to use in a CNN. In particular, adding Gaussian noise to a signal is one of the DAT that is commonly employed. 

On the other hand, Persistence Spectrum (PS), also known as Power-Spectrum Histogram, shows the percentage of the time that a particular frequency is present in a signal. That is to say that the longer a given frequency persists in a signal as it evolves, the higher is its percentage of time. Therefore, the brighter it will appear in the persistence spectrum (PS). Hence, if there is any hidden component in the signal, it will be revealed, even if it is a light one. Thus, the PS images are suitable to be used in a CNN. 

To summarize, since the use of soft starters makes it more difficult to identify the fault-related patterns with the most commonly used time–frequency tools, the main goal of this work was to obtain a methodology that led to an easier identification of the presence of rotor faults in soft-started induction motors. Additionally, the suitability of the method to perform an automatic fault classification system was also a goal of this work. Having this in mind, the characteristics of the PS made it suitable for this application, one of them being its ability to reveal very short events present in the analyzed signal. Finally, the stray-flux during the startup transient was the chosen magnitude for this study due to its richer harmonic content compared to other magnitudes.

In order to verify the effectiveness of the proposed methodology, a test-bench consisting of a 1.1 kW induction motor and a DC motor acting as a load was used. Four different commercial soft starters were used to start the motor. The obtained results, achieving an accuracy rate of 100% for each model separately and 99.89% for all the models together, show the capabilities of the proposed approach.

Finally, to provide a global idea of the paper, its structure is presented here: Section 2 exposes the materials and methods, including the theoretical background and the proposed methodology. Section 3 gives information about the experimental setup used for the tests. Section 4 shows the results and their discussion and finally, Section 5 gives the conclusions of the study.

## 2. Materials and Methods

### 2.1. Stray-Flux Analysis

In recent years, the use of the magnetic flux generated by electrical motors to obtain information about their health state has gained interest. The analysis of this magnitude has been proven to be a good alternative to other typical techniques used in the industry for the condition monitoring of electrical motors (e.g., MCSA). 

Within this methodology, two approaches have arisen: (1) air-gap flux analysis [29] and (2) stray-flux analysis [30]. Among them both, the second one has attracted a significant interest because of many reasons. Among them, the low cost of the required sensors [31] and their simple and flexible installation on the frame of the motor [20], the fact that it is a non-invasive technique [17] as well as the fact that it provides reliability in some cases where other techniques yield to false fault positives [4,32] are the most important. 

**Figure 1:**
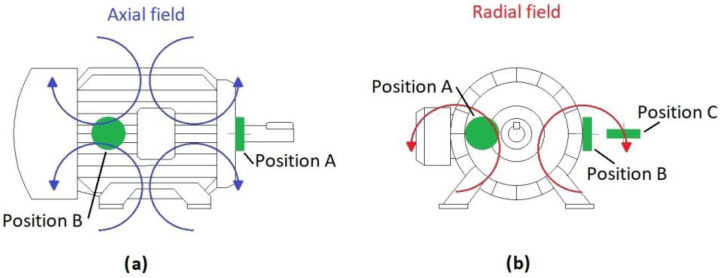
Flux sensor positions and stray-flux components: (**a**) axial field; (**b**) radial field.

Due to the non-invasive nature of the stray-flux analysis technique, it is possible to install sensors in different positions on the motor frame. This fact allows one to capture different flux components depending on the sensor position [33]. In an induction motor, axial and radial stray-flux components can be distinguished [34]. It has been proven in other works that the presence of faults in electrical motors may affect to the stray-flux, thus amplifying some specific frequency components of the stray-flux signal that depend on the existing fault [35]. In Figure 1, the different stray-flux components and positions of the sensor are shown. In this regard, in position A, mainly the axial flux is captured by the sensor, while in position C, mainly the radial component is acquired. Finally, setting the sensor in position B allows one to capture a combination of radial and axial stray-flux. 

### 2.2. Fault-Related Patterns: Theoretical Frequency Evolution during the Start-Up Transient

Many previous works have proven that the presence of rotor faults affect the stray-flux, amplifying some specific harmonics which are related to each fault. Particularly, as it has been stated by some researchers, rotor bar breakages affect the following harmonics in the Fourier spectrum of stray-flux signals:

1. Side band harmonics (*f_SH_*): These harmonics mainly appear in the radial component of the stray-flux [35,36]. Their frequency values can be calculated by Equation (1):
  (1) $$f_{SH}=f\cdot \left(1\pm 2\cdot s\right);$$
2. Axial components: Mainly observed in the axial component of the stray-flux [17], can be calculated by Equations (2) and (3):
  (2) $$f_{sf}=s\cdot f,$$
  (3) $$f_{3sf}=3\cdot s\cdot f.$$

For all the above-mentioned components, s refers to the slip and f is the supply frequency.

**Figure 2:**
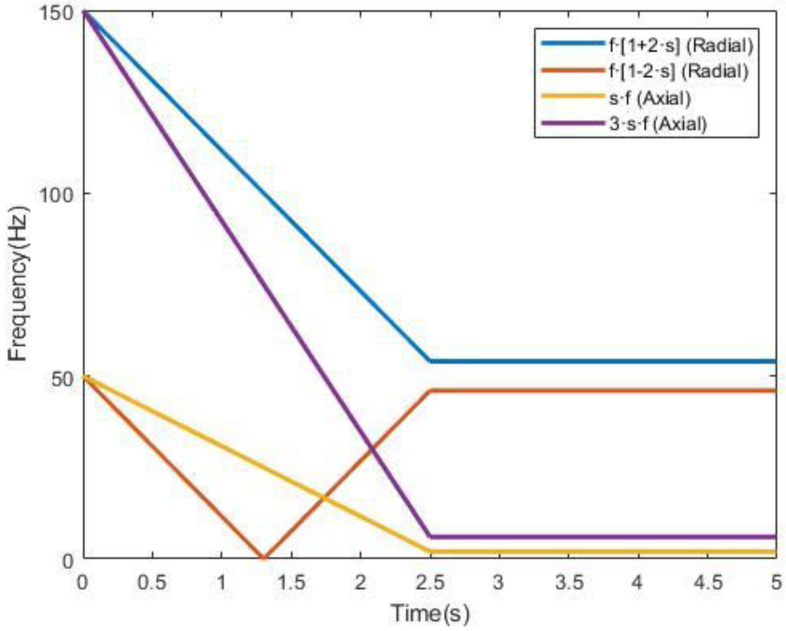
Evolution of radial and axial bar breakage-related components during a direct online start-up transient.

The theoretical transient evolutions of radial and axial components related to rotor bar breakages are shown in Figure 2. As it can be seen in that figure, the upper side harmonic, given by $f_{SH}=f\cdot \left(1+2\cdot s\right)$ and depicted in blue, drops from 150 Hz to almost 50 Hz. On the other hand, the lower side harmonic, given by $f_{SH}=f\cdot \left(1-2\cdot s\right)$ and depicted in orange, drops from 50 Hz to 0 Hz and then rises to almost 50 Hz. Regarding the axial components, s·f (depicted in yellow) drops from 50 Hz to almost 0 Hz, while 3·s·f (depicted in purple) drops from 150 Hz to almost 0 Hz. That evolution is valid for stray-flux signals during a direct online start-up transient.

### 2.3. Persistence Spectrum

Persistence Spectrum (PS) is a commonly used technique in spectrum analyzers. Also known as Spectrum Histogram, it is a histogram in power–frequency space. It allows one to see the percentage of time that a specific frequency is present in a signal. The more time a specific frequency persists in a signal as it evolves, the brighter it will appear in the PS. Therefore, it allows one to see very short events and even low power signals hidden in other signals [37].

**Figure 3:**
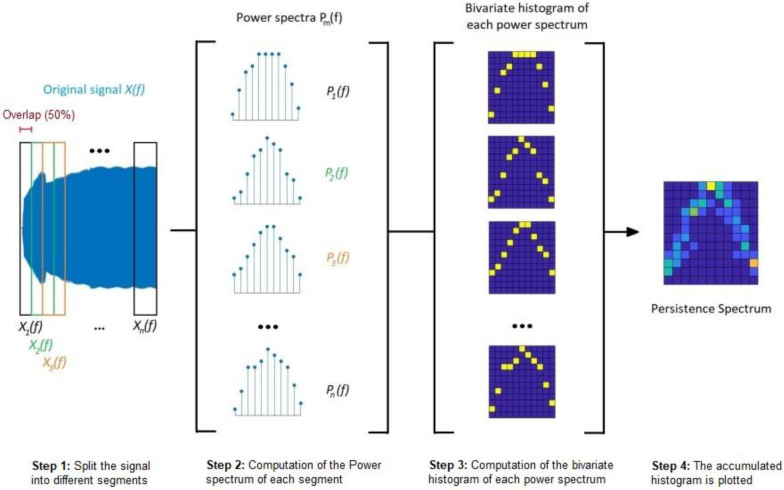
Overview of the Persistence Spectrum computation procedure. **Step 1:** Split the signal into different segments. **Step 2:** Computation of the power spectrum of each segment. **Step 3:** Computation of the bivariate histogram for each power spectrum. **Step 4:** The accumulated histogram is plotted.

The procedure to obtain the PS follows the steps listed below [37]:

- **Step 1:** The original signal is split into different segments of the same length (see Figure 3). These segments may overlap or not; but overlapping leads to more detailed spectrum analyses. The time resolution, or segment length, has to be equal to or smaller than the signal length. The number of segments, is given by Equation (4):
  (4) $$k=\left\lfloor \frac{N_{x}-L}{M-L}\right\rfloor$$
  with $N_{x}$ being the signal duration or length, L the length of the overlap and M the time resolution or segment length. Symbols ⌊ ⌋ denote a function that rounds the result to the nearest integer.
- **Step 2:** Once the signal is split, the power spectrum of each segment is computed by applying the Short-Time Fourier Transform (STFT), as shown in Figure 3. The STFT matrix is obtained by applying Equation (5).
  (5) $$X\left(f\right)=\left[X_{1}\left(f\right)\;X_{2}\left(f\right)\;\cdots X_{n}\left(f\right)\right]$$

As it was stated in [38,39,40], the *m*th element of the STFT matrix is given by Equation (6):

(6) $$X_{m}\left(f\right)=\overset{\infty }{\underset{n=-\infty }{\sum }}x\left(n\right)\cdot g\left(n-mR\right)\cdot e^{-j2\pi fn}$$

where: 

$x\left(n\right)$ = input signal at time n,

$g\left(n\right)\;$= window function (Kaiser window in this work),

$X_{m}\left(f\right)\;$= Discrete Fourier Transform (DTF) of windowed data centered in time mR,

R = number of samples between subsequent DFT (difference between segment length and overlap length).

For each segment, as stated in [39,41], the power spectrum is given by Equation (7):

(7) $$P_{m}\left(f\right)={\left|X_{m}\left(f\right)\right|}^{2}.$$

- **Step 3:** A bivariate histogram of the power spectrum logarithm is computed for each time value. In this regard, each segment corresponds to a time value. Every power–frequency bin in which there is signal energy at that time, increases the corresponding matrix element by “1” (see Figure 3).
- **Step 4:** Once all the bivariate histograms are obtained, an accumulated histogram is plotted against the frequency (*X* axis) and the power (*Y* axis). Brighter colors represent higher presence in time of a component.

In Figure 3, an overview of the Persistence Spectrum computation procedure is shown [37]. In this figure, a 50% overlap rate is applied, which is the same rate used in this work.

### 2.4. Convolutional Neural Network (CNN)

Being a type of Artificial Neural Networks (ANN), CNNs perform specially well in recognizing images. They are composed of an input layer, several hidden layers and an output layer. What differentiates CNNs from other ANNs is the presence of at least one convolutional layer as a part of the hidden layers. And it is this convolution operation which identifies local characteristics of the input data that can be used for the classification. 

The basics of CNNs are explained In [42]. Nevertheless, their way of operation is summarized here:

- Learning stage:

Assuming that the input data $x^{l-1}$ includes m 2-D matrices, they are convolved in the convolutional layer with the learnable kernels that a layer consists of. That is to say that for each input matrix $x_{i}^{l-1}\left(i\in m\right)$, it is convolved with the kernel (or filter) $k_{j}$. After this, the sum of all that is added to the bias $b^{l}$. Then, the activation function f (typically a ReLU function) is fed with the result and produces the final output of the $j_{th}$ kernel (or filter). This is mathematically expressed in Equation (8). After this, a batch normalization layer is typically used. It helps to make the training faster by normalizing every input channel across a mini batch. Finally, a pooling layer divides the input into smaller areas and then calculates the average or the maximum of that areas [43].

(8) $$x_{j}^{l}=f\left(\underset{i\in M_{j}}{\sum }x_{i}^{l-1}*k_{j}^{l}+b_{j}^{l}\right)$$

- Classification stage:

Consisting typically of two layers (fully connected layer and classification layer), this stage combines all the features extracted from the input data in the learning stage. First, the fully connected layer generates a vector with as much dimensions as the number of classes the CNN is able to predict. Then, a classification layer, usually using a softmax function, provides the classification output.

### 2.5. Proposed Methodology

The proposed methodology consists of 5 main steps. First, the current and stray-flux signals are captured by means of different sensors. Then, White Gaussian Noise is added to the original signal to increment the number of signals. In the third step, the persistence spectrum of each signal is computed. Then, the PS images obtained are cropped and resized to adapt them to the requirements of the CNN, and finally, these images are used as input of the classification CNN in the fifth step. Stated yet another way, the input of the CNN will be the PS images after being cropped and resized to 224 × 224 × 3 images, while the output of the CNN will be the three rotor fault classes, namely healthy state, one broken bar and two broken bars.

**Figure 4:**
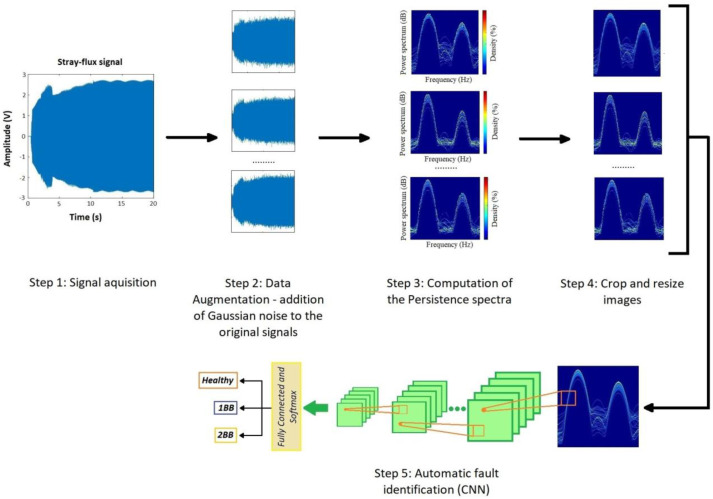
Proposed methodology overview.

To better illustrate the sequence of the procedure, a flux diagram of the proposed methodology is shown in Figure 4:

As mentioned before, the proposed methodology follows the steps listed here:

- **Step 1: Acquisition of the current and stray-flux signals.** These two magnitudes were captured, simultaneously, during the start-up transients. To do this, a current clamp and a coil-based flux sensor, both of them described in the next section, were used. The signals were stored in a waveform recorder (oscilloscope) and then downloaded to a PC, where the signal analyses were performed. The selected position of the flux sensor was the one allowing one to capture a combination of radial and axial stray-flux (Position B, see Figure 1).
- **Step 2: Data augmentation**. In order to generate a higher number of training samples, a Data Augmentation Technique (DAT) was applied. In this case, the addition of White Gaussian Noise (WGN) to the original stray-flux signals was the selected technique.

**Figure 5:**
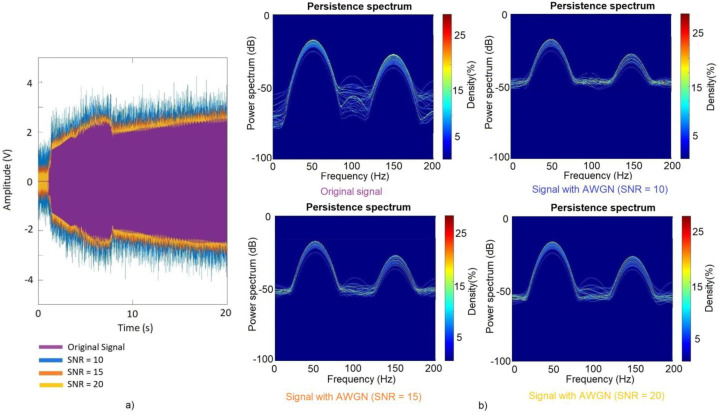
(**a**) Original stray-flux signal and different signals with AWGN overlapped. (**b**) Persistence Spectra of the original signal and the three signals with different AWGN.

The addition of Gaussian Noise to the original signals is a data augmentation tool that is frequently used. This technique can increase the dataset by selecting different values of standard deviations (σ) [44] or, since it directly affects the value of σ, different values of Signal-to-Noise Ratio (SNR). In this regard, also in [44], it is proven that values of SNR smaller than 10 dB report low improvements to the accuracy of the classification methods. On the other hand, authors in [45] pointed out that large ranges of SNR in the injected noise allow one to obtain better performance of the test datasets. In other works, as in [46], the authors set the SNR range for the injected noises between 10 dB and 20 dB. Taking all this into account and also that a level of SNR of 20 dB is commonly considered as a good value of AWGN in electrical signals [19], a set of Gaussian Noises with SNR from 10 dB to 20 dB, in steps of 0.2 dB, was performed for this work. Thus, the number of signals of the dataset, including the original ones, reached the values shown in Table 1:

In Figure 5, a comparison between one original stray-flux signal and three of the signals with AWGN is shown. In addition, the Persistence Spectrum computed for each of the mentioned signals are shown.

**Figure 6:**
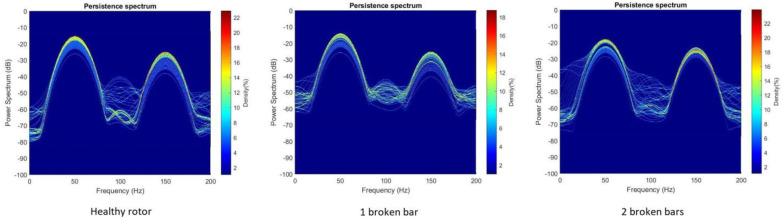
PS images for the ABB model, with nominal load and with the time-ramp set in 20 s and initial voltage set in 40%, for the three health cases: Healthy, one broken bar and two broken bars. All obtained from the captured stray-flux signal.

- **Step 3: Computation of the Persistence Spectrum of each signal.** Once all the stray-flux signals were obtained, both the captured ones and those resulting from the data augmentation process, the start-up transient was identified and isolated from the signal itself. Then, the Persistence Spectrum (PS) was computed for all the transients obtained, setting an overlap of 50% and using the Kaiser window as window function. The process to obtain the PS was the one referred in Section 2.3. As a result, a set of 3780 images was obtained, one for each transient. Those images were stored in different folders. For each model of soft starter, the images were divided into three folders depending on the health state of the rotor (namely healthy, one broken bar and two broken bars). Those folders contained, in each case, the resulting PS images for the different parameter settings of the soft starter model, with and without load. In Figure 6, an example of the PS images obtained is shown.

**Figure 7:**
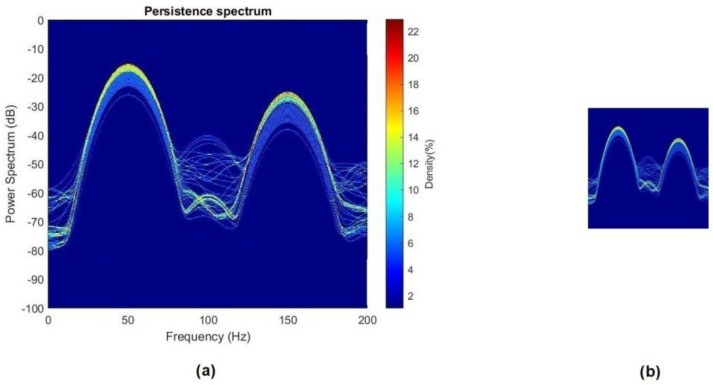
(**a**) PS image; (**b**) PS image cropped and resized.

**Figure 8:**
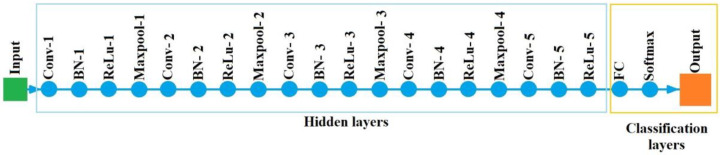
Details of the CNN.

**Figure A1:**
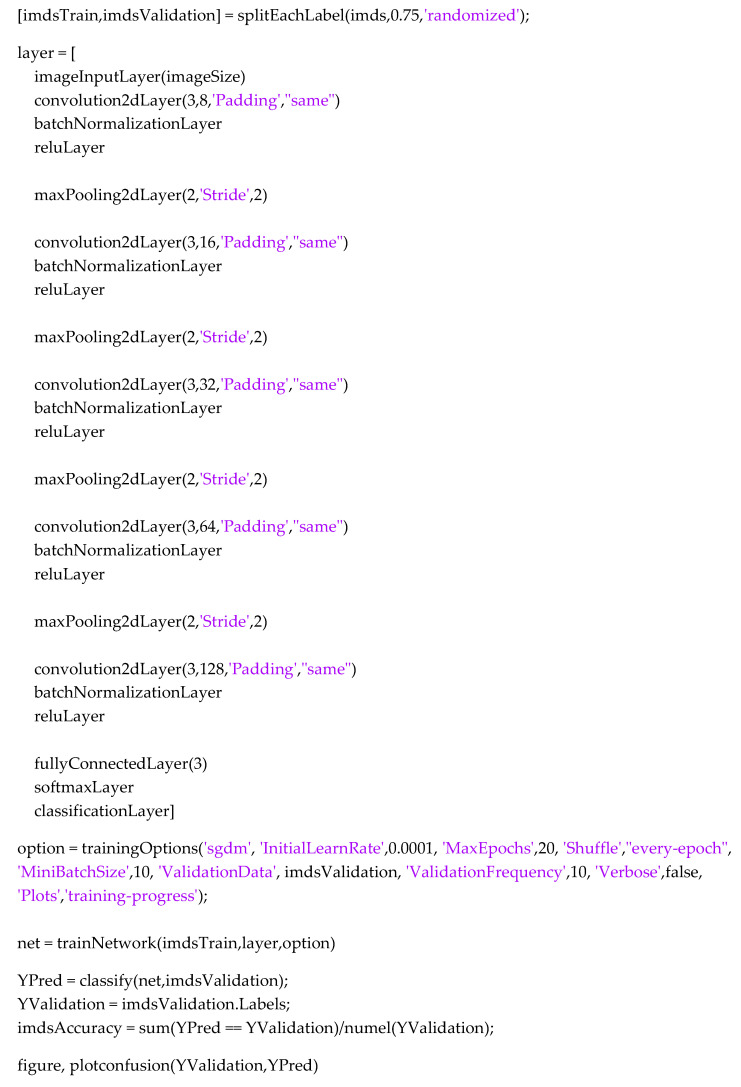
Matlab pseudocode of the CNN.

As explained in Section 2.3, the Persistence Spectrum represents, by means of a color map, the percentage of time that a particular frequency appears in a signal. The *x*-axis shows the frequency (in Hz) and the *y*-axis the power (in dB). Thus, it is a time–frequency view. In this regard, the representing limits for the frequency were set between 0 Hz and 200 Hz, while for the power, between −100 dB and 0 dB. Those limits were chosen due to the following reasons:

- Regarding the power, the limits were set attending to the maximum and minimum value obtained from all the Persistence Spectra computed for all the signals.
- Regarding the frequency, the limits were set attending to the frequencies where the components related to the patterns of the studied fault (broken bars) must appear.

- **Step 4. Crop and resize images.** In order to adapt the PS images obtained in the previous step to the needs of the CNN, they were cropped and resized. The main aim for the cropping was to eliminate the color bar and the axis legends, keeping only the area where the PS was represented. On the other hand, since the CNN input size for the images was set in 224 × 224 pixels, the cropped images needed to be reduced to that size. In Figure 7, an example of the cropped and resized images against the PS images can be seen.
- **Step 5. Automatic fault identification (CNN).** For the automatic classification of the different health states of the rotor (healthy, 1 BB and 2 BB), a self-developed Convolutional Neural Network (CNN) was used. It was implemented in MATLAB platform, and the detailed information of the CNN layers is shown in Figure 8 and Table 2. Additionally, the MATLAB pseudocode is shown in Appendix A, Figure A1.

With regards to the training process, the Stochastic Gradient Descent with Momentum algorithm was selected. The initial learning rate was set in $10^{-4}$, the momentum in 0.9 and the $L_{2}$ regularization factor (or weight decay factor) in $10^{-4}$. Furthermore, the min-Batch size was set in 10, attending to the results available in the technical literature. For instance, in [47], it is stated that sizes smaller than 32 allow one to obtain better training stability and generalization results. By their side, authors in [48] say that values above 10 allow faster computations. Finally, the maximum number of epochs was set in 20. The number of validation samples during the training was 25% of the available samples, randomly selected. An overview of the properties is shown in Table 3.

## 3. Experimental Setup

**Figure 9:**
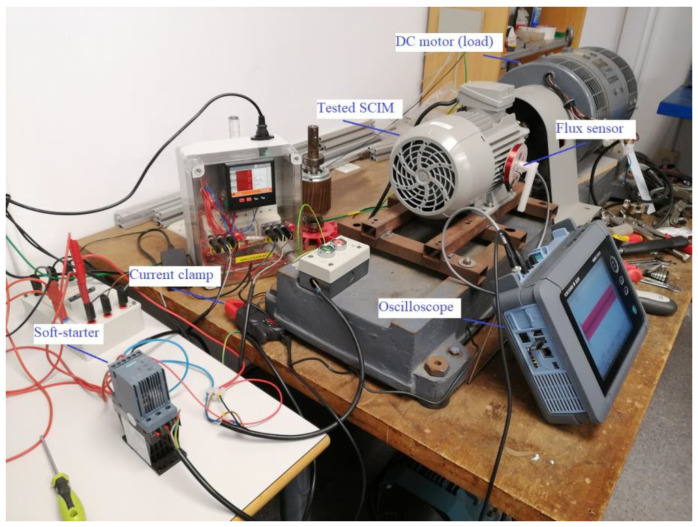
Test-bench used in the laboratory.

**Figure 10:**
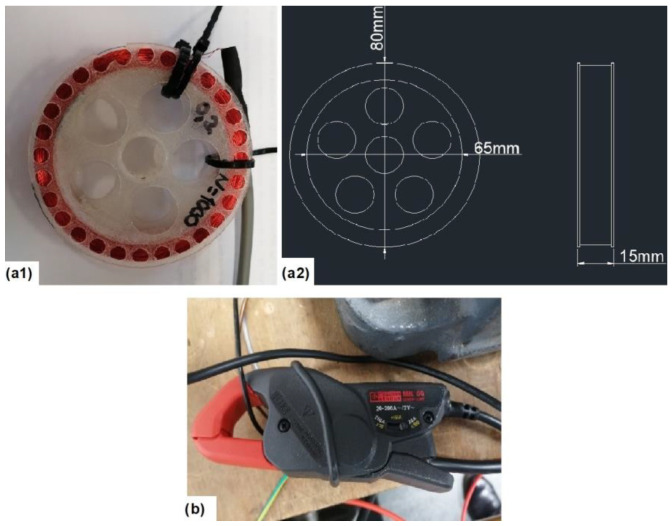
Sensors employed: (**a1**) Handmade coil-flux sensor. (**a2**) Dimensions and shape of the coil sensor. (**b**) Current clamp.

In order to validate the effectiveness of the proposed methodology, several tests were carried out in the laboratory. The test-bench employed was the one shown in Figure 9. It consisted of a 1.1 kW squirrel cage induction motor (tested motor), coupled to a DC motor which acted as a load. The tested motor (SCIM) was started by means of four different commercial soft starters. During every start-up, the stray-flux and the current demanded by the motor were captured. To capture the stray-flux, a handmade coil-based sensor attached to the motor frame was used. A picture of it and its shape and main dimensions are shown in Figure 10(a1,a2), respectively. To capture the current signal of one of the supply phases of the motor, a current clamp was used (see Figure 10b). All these signals were recorded with an oscilloscope and then downloaded to a PC, where the signal analyses were performed. Both the stray-flux and the current signals were acquired for 40 s at a sampling rate of 5 kHz. All the analyses and training and validation processes were conducted on a PC, with an Intel Core i5-9400 1 CPU (2.9 GHz) and 8 GB of memory.

The main characteristics of the tested motor (SCIM) are shown in Table 4.

The main characteristics of the coil-flux sensor are the ones listed in Table 5. In addition, as said before, in Figure 10(a1, a2)), the shape and dimensions of the coil sensor can be seen.

On the other hand, the main characteristics of the current clamp are listed in Table 6 and a picture of it is shown in Figure 10b.

**Figure 11:**
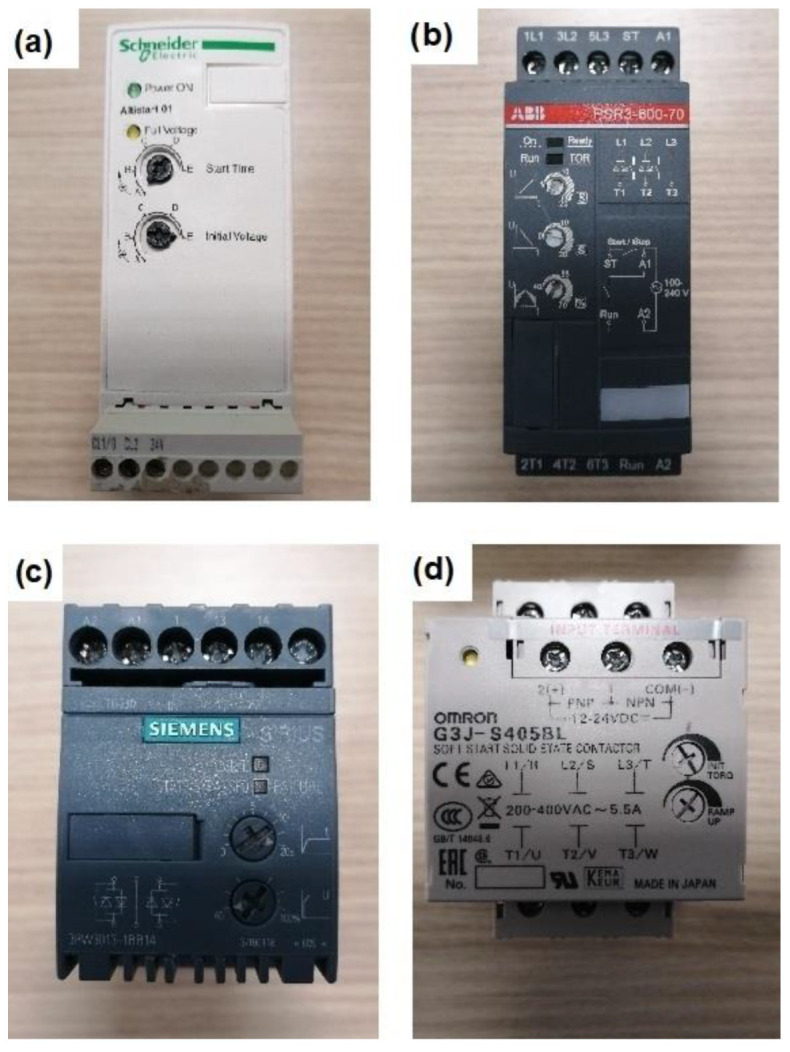
Models of tested soft starters: (**a**) Schneider ALTISTART 01; (**b**) ABB PSR3-600-70; (**c**) Siemens SIRIUS 3RW3013-1BB14; (**d**) Omron G3J-S405BL.

To carry out all the tests, four different models of soft starters were employed. Each of them had different topologies, controlling one-, two- or the three-supply phases depending on the model. Furthermore, each model allowed one to control the start-up time-ramp and the initial voltage or torque. The different models of soft starters used for the tests were the ones shown in Figure 11, and their main characteristics are listed in Table 7.

**Figure 12:**
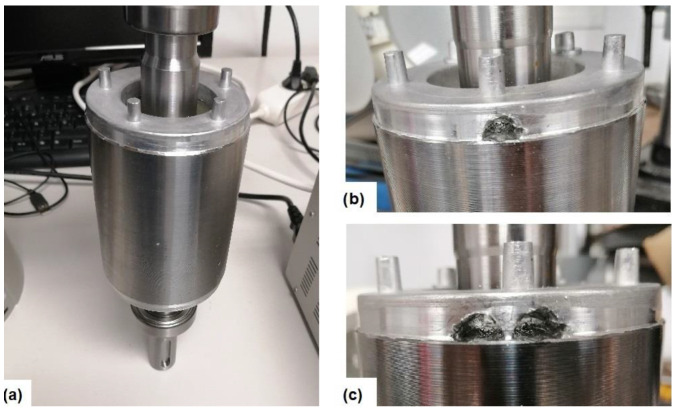
Detail of the rotor in the different health states: (**a**) Healthy. (**b**) One broken bar. (**c**) Two broken bars.

Regarding the studied fault, different bar breakages were induced in the rotor of the SCIM tested. Firstly, once the healthy rotor was tested, one rotor bar was broken by drilling a hole in the junction with the end short-circuit ring. Then, once the one-broken-bar tests were carried out, a second rotor bar, contiguous to the previous, was broken in the same way. A detail of the healthy rotor and the bar breakages forced is shown in Figure 12.

The tests were carried out following the same sequence for the four models of soft starters. First, the healthy motor was started, without load, by means of one of the soft starters. Different combinations of time-ramp and initial voltage/torque were performed for each model of soft starter and for each of those combinations, the tested motor was started once. Then, the same tests were repeated, but this time with the tested motor fully loaded. This was achieved by varying the excitation voltage of the DC machine coupled to the tested motor. Afterwards, the procedure was repeated first for the case of one broken bar and then for the case of two broken bars.

For each start-up, the coil-flux sensor was placed in a position which allowed one to obtain a combination of axial and radial flux (called Position B, see Figure 1). In addition, the current signals of one of the supply phases was captured by means of the above-mentioned current clamp. These tests allowed one to obtain a batch of signals of the tested SCIM under different starting conditions. The different combinations of parameters performed for each soft starter are listed in Table 8, and also the number of signals obtained for each model.

## 4. Results and Discussion

In this section, the results obtained by applying the proposed methodology are shown. First, a comparison of the different persistence spectra for the four models of soft-starter and each health case are presented, highlighting the differences found. Then, the effectiveness of the CNN proposed for each model of soft starter separately and for all of them combined is shown. 

**Figure 13:**
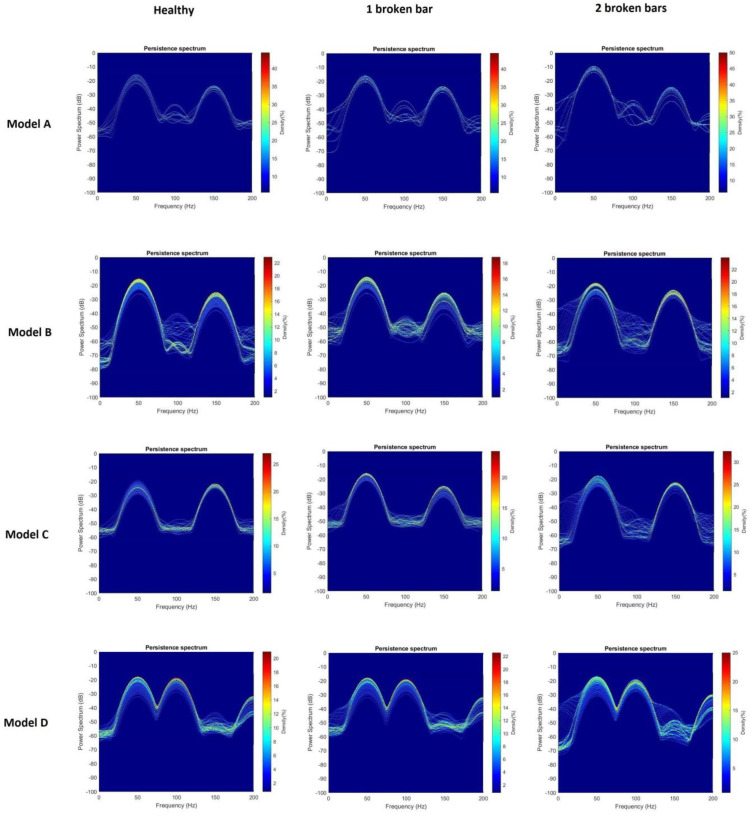
Comparison of Persistence Spectrum images for the three rotor health cases, for each model of soft starter.

Although many analyses were carried out in this work, only the most representative are shown here. In this regard, in Figure 13, persistence spectra for each rotor health state and soft starter model are compared. Those persistence spectra correspond to tests when the motor was fully loaded. The settings for each soft starter model, were those corresponding to the combination of longest time-ramp and lowest initial voltage (see Table 8).

Taking a look at the images in Figure 13, some differences can be distinguished. For all the cases, some components from 0 Hz to 50 Hz increment their amplitudes as the fault worsens. This also happens to some components from 150 Hz to 50 Hz. This fact fits with the typical behavior of the axial and radial components associated to the presence of broken bars. As it can be seen in Figure 2, axial components s·f and 3·s·f evolve from 50 Hz to almost 0 Hz for the first one and from 150 Hz to almost 0 Hz for the second one. On the other hand, radial components evolve from 50 Hz to 0 Hz and again to almost 50 Hz for the case of $f\cdot \left(1-2\cdot s\right)$ and from 150 Hz to almost 50 Hz for the case of $f\cdot \left(1+2\cdot s\right)$. Since the position of the flux sensor allowed one to capture the combination of radial and axial stray-flux, it makes sense to see the influence of both types of components in persistence spectra.

**Figure 14:**
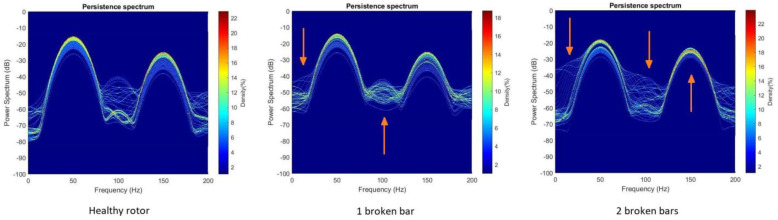
Example of differences in PS for each health state of the rotor.

In Figure 14, as an example, the above-mentioned differences for each health state of the rotor are highlighted in a set of PS images. The same differences can be identified in all the cases studied.

**Figure 15:**
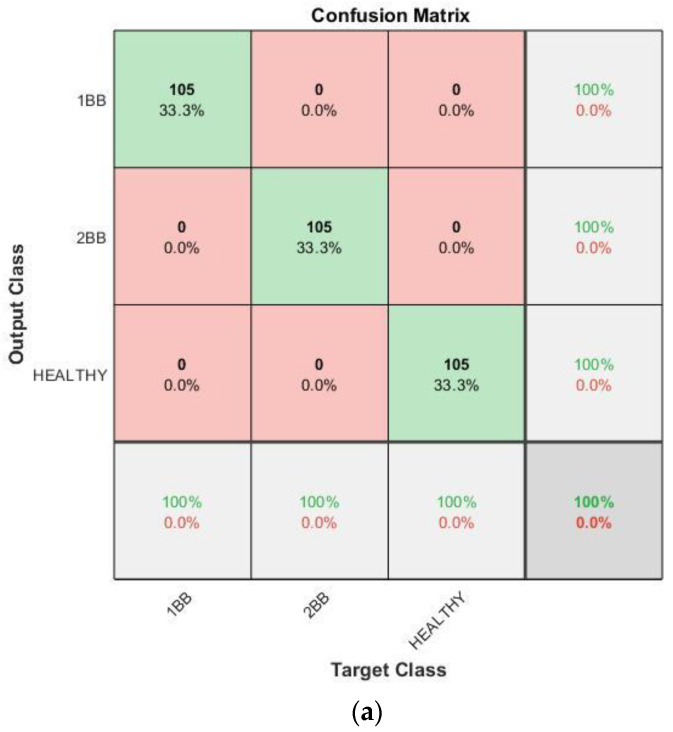

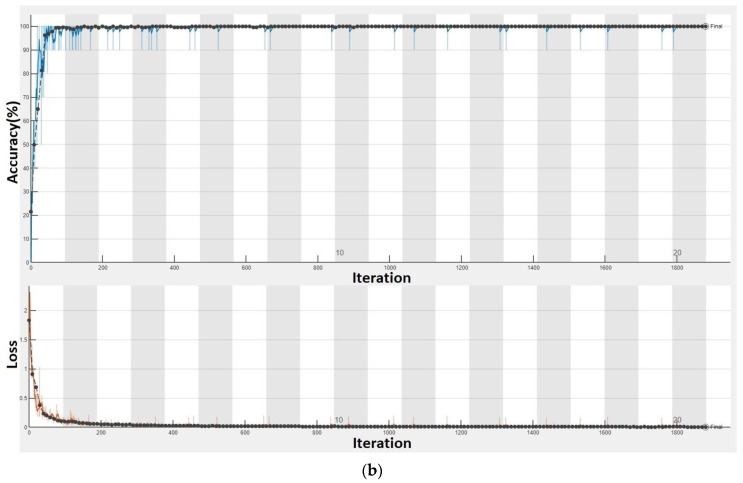
Results for Schneider soft starter: (**a**) Confusion matrix. (**b**) Training progress.

**Figure 16:**
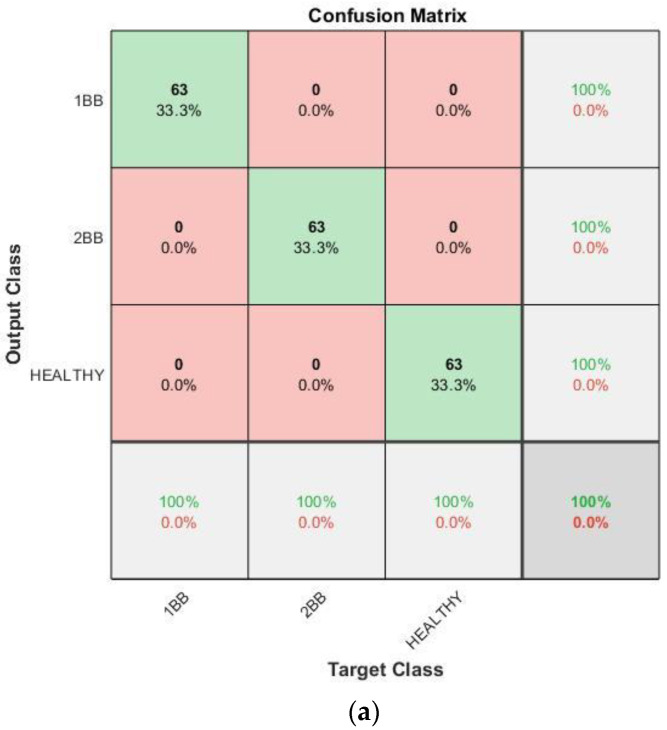

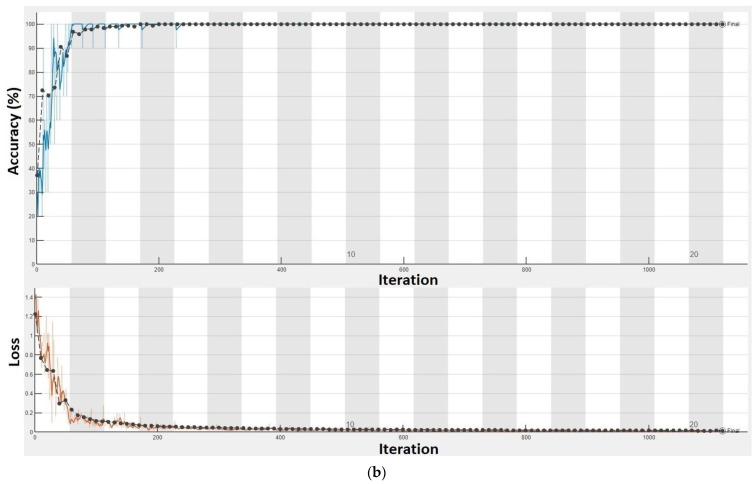
Results for ABB soft starter: (**a**) Confusion matrix. (**b**) Training progress.

**Figure 17:**
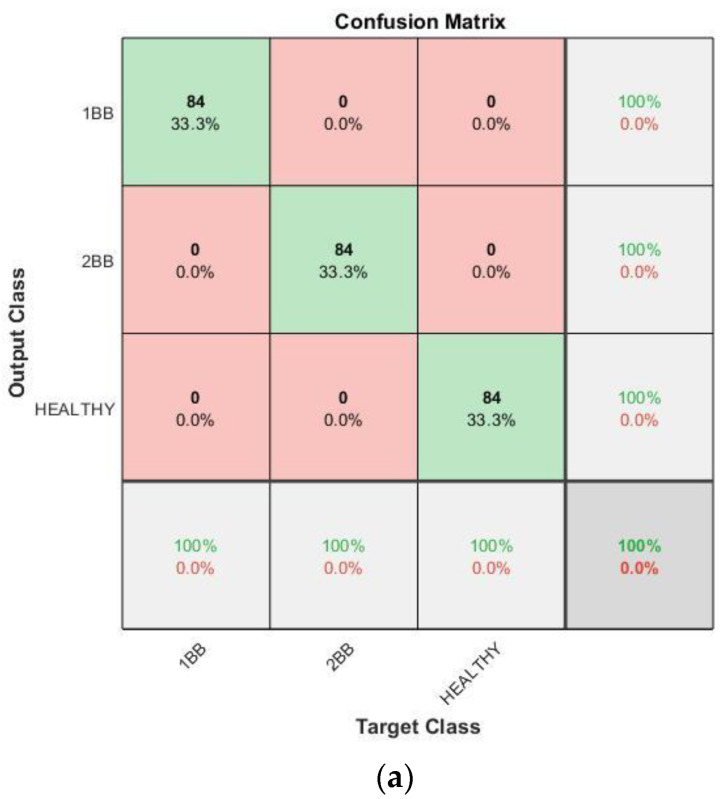

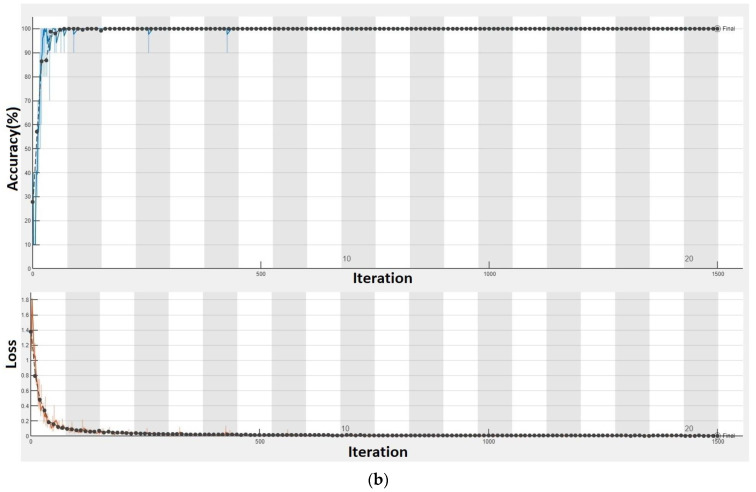
Results for Siemens soft starter: (**a**) Confusion matrix. (**b**) Training progress.

**Figure 18:**
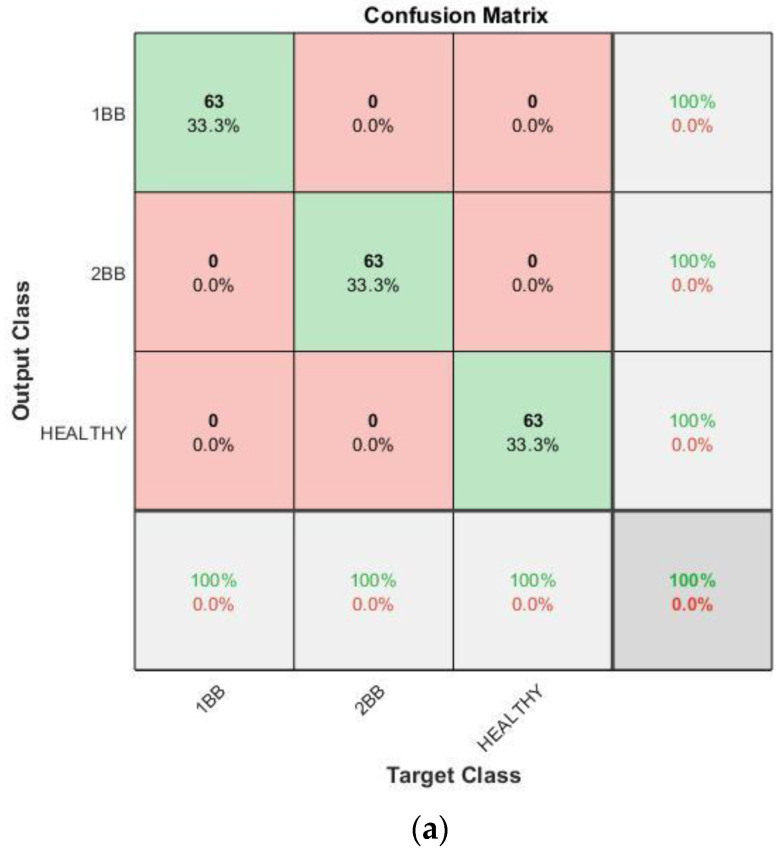

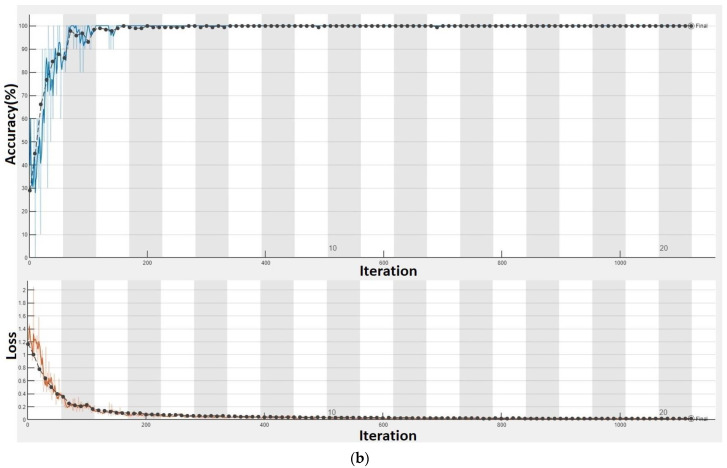
Results for Omron soft starter: (**a**) Confusion matrix. (**b**) Training progress.

With regards to the effectiveness of the proposed methodology, Figure 15, Figure 16, Figure 17 and Figure 18 show the confusion matrices and the training progresses for each model of soft starter, separately.

For the case of the SCHNEIDER model, 945 training samples (315 samples per category) were used to train the CNN and 315 different samples (105 samples per category) were used for the validation. In Figure 15, it can be observed that the accuracy of the CNN reaches 100%, which means that the methodology can identify and separate the different rotor health states in every case, even with different combinations of time-ramp and initial voltage. In addition, the training process reaches 100% accuracy after about 150 iterations, in epoch two. Moreover, it becomes stable at 100% after, more or less, 900 iterations.

For the case of the ABB model, 567 training samples (189 samples per category) were used to train the CNN and 189 different samples (63 samples per category) were used for the validation. In Figure 16. it can be observed that the accuracy of the CNN also in this case reaches 100%. Furthermore, the training process reaches 100% accuracy after about 180 iterations, in epoch four. Moreover, it becomes stable at 100% after, more or less, 200 iterations.

For the case of the SIEMENS model, 756 (252 samples per category) training samples were used to train the CNN and 252 different samples (84 samples per category) were used for the validation. In Figure 17, it can be observed that the accuracy of the CNN also in this case reaches 100%. In addition, the training process reaches 100% accuracy after about 68 iterations, in epoch one. Moreover, it becomes stable at 100% after, more or less, 160 iterations.

For the case of the OMRON model, 567 training samples (189 samples per category) were used to train the CNN and 189 (63 samples per category) different samples were used for the validation. In Figure 18, it can be observed that the accuracy of the CNN also in this case reaches 100%. Furthermore, the training process reaches 100% accuracy after about 160 iterations, in epoch three. Moreover, it becomes stable at 100% after, more or less, 340 iterations.

**Figure 19:**
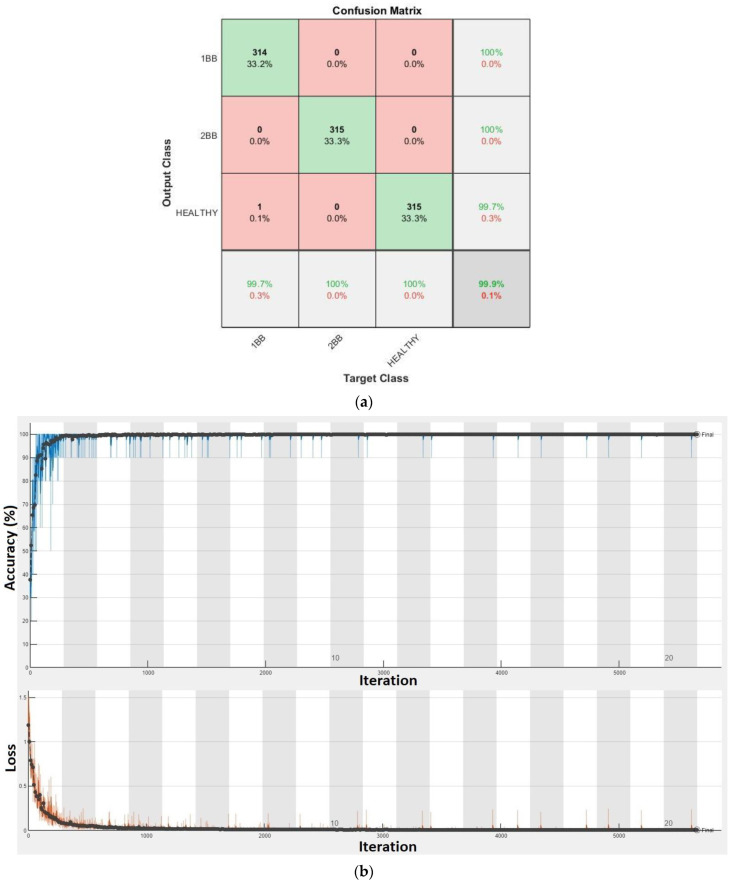
Results for all the models of soft starter combined: (**a**) Confusion matrix. (**b**) Training progress.

Finally, in Figure 19, the confusion matrix and the training progress for all the models of soft starters combined are shown. In this case, 2835 training samples (945 samples per category) were used to train the CNN and 945 (315 samples per category) different samples were used for the validation. Although four different topologies of soft starter and different combinations of time-ramp duration and initial voltage were compared in this case, the accuracy achieved a rate of 99.89%. That is to say that only one of the samples was misclassified. Moreover, the misclassified prediction was among the healthy and first stage of failure (one broken bar). The training process reaches the referred accuracy after about 650 iterations, in epoch three, and it becomes stable at 99.89% after, more or less, 3000 iterations.

Once the capabilities of the proposed methodology have been exposed, in Table 9, it is compared with the results of other methodologies proposed for broken bar automatic detection in soft-started induction motors. Additionally, since there are not many works focused on soft starters, the results of other works focused on Direct Online starting are also included in the table. 

With regards to the accuracy of the methodologies, some of the works in Table 9 achieved a rate of 100% [22,23,49], but all of them were focused on DOL starting, and they were analyzing current signals. On the other hand, those works focused on soft-started induction motors and achieved, in both cases, an overall accuracy of 94.40%, analyzing the stray-flux [27] and the combination of stray-flux and current [28]. Both of them relied on the STFT as the time–frequency analysis tool, which displays noisy time–frequency maps when soft starters are used, making it more difficult to identify the typical patterns related to broken bars.

On the contrary, the proposed methodology relies on the use of Persistence Spectrum as time–frequency display. This method allows one to see even very short events, leading to an easier identification of fault-related patterns and allowing one to achieve an accuracy rate of 100% when analyzing each model of soft starter separately and 99.89% when analyzing all the models combined.

## 5. Conclusions

In this work, a novel methodology to automatically detect and categorize the severity of rotor faults in induction motors driven by soft starters is presented. This methodology relies on the computation of the Persistence Spectrum of the start-up transient of stray-flux signals. Then, the images obtained are used as input for a self-developed CNN in order to obtain their classification. Experimental results prove that not only the accuracy achieved is very high, improving the ones of other works focused on soft-started induction motors, but also the convergence of the training progress to the final accuracy rate is very fast.

Thus, taking into account all the above-mentioned and the results shown in the previous section, the following conclusions arise:

- The use of the persistence spectrum as a way to analyze the stray-flux signals during the start-up transient allows one to detect the health state of the rotor.
- Even in the case analyzed in this paper, where soft starters are used to drive the motor and so the level of noise in the signal makes it difficult to identify the characteristic patterns of the fault when using typical time–frequency tools (such as STFT), the use of this method allows one to identify not only the presence of the fault, but also its severity.
- Even when different starting settings are performed and different topologies of soft starters are used, this method achieves a very high accuracy rate (99.89%), proving its reliability.
- This method is a promising way to diagnose induction motors when using soft starters and could lead to integrate the diagnosis system in the soft starter itself, only by adding an external flux sensor.

The results prove that the use of this method could lead to a reliable diagnosis of the health state of the rotor of SCIMs, allowing one to schedule proper maintenance and hence, reducing the energy consumption due to the running of damaged motors and avoiding unscheduled shutdowns of the processes depending on them.

Finally, although a very high accuracy has been achieved with this classification method, further studies have to be carried out in order to evaluate the generalized possibilities of the proposed methodology. The authors are carrying out more tests to evaluate the application of this method to other faults and SCIMs of different nominal power. Furthermore, the authors plan to evaluate the application of this method to other types of motors, like synchronous reluctance motors or permanent magnet synchronous motors, to detect other kind of failures, as well as proposing complementary methodologies based on computing statistical indicators based on the obtained results that may enhance the diagnosis in some specific cases.

## Figures and Tables

**Table 1:** Number of signals after data augmentation, including the original ones.

Soft-Starter	Healthy	1 BB	2 BB
Schneider	420	420	420
ABB	252	252	252
Siemens	336	336	336
Omron	252	252	252
Total	1260	1260	1260

**Table 2:** Detailed information of the CNN layers.

No.	Layer Type	Layer Activation Size	Layer Parameters
1	Input	224 × 224 × 3	PS images
2	Convolution (Conv-1)	224 × 224 × 8	FS: 3; No. F: 8; Stride: 1; Padding: same
3	Batch Normalization (BN-1)	224 × 224 × 8	8 channels
4	ReLu-1	224 × 224 × 8	-------
5	Max pooling-1	112 × 112 × 8	Pool size: 2; Stride: 2
6	Convolution (Conv-2)	112 × 112 × 16	FS: 3; No. F: 16; Stride: 1; Padding: same
7	Batch Normalization (BN-2)	112 × 112 × 16	16 channels
8	ReLu-2	112 × 112 × 16	-------
9	Max pooling-2	56 × 56 × 16	Pool size: 2; Stride: 2
10	Convolution (Conv-3)	56 × 56 × 32	FS: 3; No. F: 32; Stride: 1; Padding: same
11	Batch Normalization (BN-3)	56 × 56 × 32	32 channels
12	ReLu-3	56 × 56 × 32	-------
13	Max pooling-3	28 × 28 × 32	Pool size: 2; Stride: 2
14	Convolution (Conv-4)	28 × 28 × 64	FS: 3; No. F: 64; Stride: 1; Padding: same
15	Batch Normalization (BN-4)	28 × 28 × 64	64 channels
16	ReLu-4	28 × 28 × 64	-------
17	Max pooling-4	14 × 14 × 64	Pool size: 2; Stride: 2
18	Convolution (Conv-5)	14 × 14 × 128	FS: 3; No. F: 128; Stride: 1; Padding: same
19	Batch Normalization (BN-)	14 × 14 × 128	128 channels
20	ReLu-5	14 × 14 × 128	-------
21	Fully connected (FC)	1 × 1 × 3	Output size: 3
22	Softmax	1 × 1 × 3	-------
23	Output	-------	Fault class

FS: Filter Size; No. F: Number of filters.

**Table 3:** Training properties of the CNN.

Training Algorithm	Stochastic Gradient Descent with Momentum (SGDM)
Momentum	0.9
Initial learning rate	0.0001
Weight decay factor (*L*_2_)	0.0001
Mini-batch size	10
Maximum epochs	20
Validation samples	25%

**Table 4:** Rated characteristics of the tested motor (SCIM).

Frequency (Hz)	50
Power (kW)	1.1
Rated Current (A)	2.4
Rated Voltage (V)	400
Connection	Star
Pole Pairs	2
Rated Speed (rpm)	1440
Number of Rotor Bars	28

**Table 5:** Main characteristics of the handmade coil-based flux sensor.

Number of Turns	1000
Wire Diameter (mm)	0.2
External Diameter (mm)	80
Internal Diameter (mm)	65
Width (mm)	15

**Table 6:** Main characteristics of the current clamp.

Manufacturer	Chauvin Arnoux
Model	MN 60
Current range (A AC)	20–200
Maximum output signal (V AC)	2
Rated Voltage (V)	600 V (CAT III)–300 V (CAT IV)
Bandwidth (kHz)	0.04–40
Output	Coaxial cable with insulated BNC connector

**Table 7:** Main characteristics of the four models of soft starter used.

	Model A	Model B	Model C	Model D
Manufacturer	Schneider	ABB	Siemens	Omron
Country of Production	Germany	China	Germany	Japan
Model	ATS01N109FT	PSR3-600-70	3RW3013-1BB14	G3J-S405BL
Rated Power (kW)	4	1.5	1.5	2.2
Rated Voltage (V)	400	380–400	380–400	380–400
Rated Frequency (Hz)	50	50	50	50
Maximum Current (A)	9	3.9	3.6	5.5
Voltage Ramp Duration (s)	1–5	1–20	0–20	1–25
Controlled Phases	R	R-S	R-T	R-S-T

**Table 8:** Parameter combinations performed for each soft starter. Number of signals obtained.

MODEL A: Schneider	Combination 1	Combination 2	Combination 3	Combination 4	Combination 5	Signals
Time-ramp Duration (s)	1	2	3	4	5	
Initial Voltage (%)	80	67.5	55	40.5	30	
Total number of signals						10
**MODEL B: ABB**	**Combination 1**	**Combination 2**	**Combination 3**			**Signals**
Time-ramp Duration (s)	1	10	20			
Initial Voltage (%)	70	55	40			
Total number of signals						6
**MODEL C: Siemens**	**Combination 1**	**Combination 2**	**Combination 3**	**Combination 4**		**Signals**
Time-ramp Duration (s)	0	5	10	20		
Initial Voltage (%)	100	70	50	40		
Total number of signals						8
**MODEL D: Omron**	**Combination 1**	**Combination 2**	**Combination 3**			**Signals**
Time-ramp Duration (s)	1	12.5	25			
Initial Voltage (%)	72	58	44			
Total number of signals						6

**Table 9:** Proposed methodology compared with other methodologies used for automatic detection of rotor bar breakages.

Reference	Methodology	Start-Up Method	Magnitude Analyzed	Accuracy Rate
Pasqualotto et al. [18]	CNN, STFT, Data Augmentation Techniques	DOL	Stray-Flux	66.70%
Zamudio et al. [21]	FFNN, STFT	DOL	Stray-Flux and Current	95.00%
Zamudio et al. [20]	FFNN, STFT	DOL	Stray-Flux	97.00%
Rivera et al. [19]	Tooth-FFT, Pearson Correlation	DOL	Current	97.50%
Ince et al. [24]	CNN, Back-Propagation Algorithm	DOL	Current	97.87%
Camarena et al. [50]	Pearson Correlation, Wavelet Transform	DOL	Current	99.00%
Valtierra et al. [22]	CNN, STFT	DOL	Current	100.00%
Lopez et al. [49]	Normal-Distribution, Otsu Segmentation, Multi-STFT	DOL	Current	100.00%
Pasqualotto et al. [27]	CNN, STFT and Data Augmentation Techniques	Soft Starters	Stray-Flux	94.40%
Navarro et al. [28]	STFT, FFNN, Arithmetic Mean and Maximum Value	Soft Starters	Stray-Flux and Current	94.40%
**Proposed Methodology**	**CNN, Persistence Spectrum and Data Augmentation Techniques**	**Soft Starters**	**Stray-Flux**	**100.00%**

## Data Availability

Not applicable.

## Abbreviations

The following abbreviations are used in this manuscript:

IMs	Induction Motors
SCIM	Squirrel Cage Induction Motors
STFT	Short Time Fourier Transform
FFT	Fast Fourier Transform
DWT	Discrete Wavelet Transform
PS	Persistence Spectrum
CNN	Convolutional Neural Network
DAT	Data Augmentation Techniques
WGN	White Gaussian Noise
AWGN	Added White Gaussian Noise
DOL	Direct Online
MCSA	Motor Current Signature Analysis
DFT	Discrete Fourier Transform
ANN	Artificial Neural Network

## Appendix A

In this appendix, the pseudocode of the CNN is shown in Figure A1:
